# Neuroprotective Assessment of *Moringa oleifera* Leaves Extract against Oxidative-Stress-Induced Cytotoxicity in SHSY5Y Neuroblastoma Cells

**DOI:** 10.3390/plants10050889

**Published:** 2021-04-28

**Authors:** Farah J. Hashim, Sukanda Vichitphan, Patcharee Boonsiri, Kanit Vichitphan

**Affiliations:** 1 Graduate School, Khon Kaen University, Khon Kaen 40002, Thailand; farahjabbarhashim@kkumail.com; 2 Department of Biotechnology, Faculty of Technology, Khon Kaen University, Khon Kaen 40002, Thailand; sukanda@kku.ac.th; 3 Department of Biotechnology, College of Science, University of Baghdad, Baghdad 10071, Iraq; 4 Fermentation Research Center for Value Added Agricultural Products (FerVAAP), Khon Kaen University, Khon Kaen 40002, Thailand; 5 Department of Biochemistry, Faculty of Medicine, Khon Kaen University, Khon Kaen 40002, Thailand; patcha_b@kku.ac.th

**Keywords:** *Moringa oleifera*, ROS, H_2_O_2_, oxidative stress, superoxide anion, SHSY5Y neuroblastoma cell line

## Abstract

The current trend worldwide is searching plant extracts towards prevention of neurodegenerative disorders. This study aimed to investigate the neuroprotective effect of *Alpinia galanga* leaves (ALE), *Alpinia galanga* rhizomes (ARE), *Vitis vinifera* seeds (VSE), *Moringa oleifera* leaves (MLE), *Panax ginseng* leaves (PLE) and *Panax ginseng* rhizomes (PRE) ethanolic extracts on human neuroblastoma (SHSY5Y) cells. The 1-diphenyl-1-picrylhydrazyl (DPPH) radical scavenging of VSE and MLE were 81% and 58%, respectively. Ferric-reducing antioxidant power (FRAP) of ALE and MLE (33.57 ± 0.20 and 26.76 ± 0.30 μmol Fe(ΙΙ)/g dry wt., respectively) were higher than for the other extracts. Liquid chromatography coupled to quadrupole time-of-flight mass spectrometry (LC-QTOF/MS) revealed MLE active compounds. Intracellular study by nitro-blue tetrazolium (NBT) test showed that MLE and VSE had high O_2_^−^ scavenging (0.83 ± 0.09 vs. 0.98 ± 0.08 mg/mL, respectively). MLE had the highest ROS scavenging followed by PRE (0.71 ± 0.08 vs. 0.83 ± 0.08 mg/mL, respectively), by 2,7-dichlorodihydrofluorescein diacetate (DCFHDA) assay. The 3-(4,5-dimethylthiazol-2-yl)-2,5-diphenyltetrazolium bromide (MTT) cytotoxicity and neuroprotection tests on SHSY5Y showed that PRE had a better neuroprotective effect but higher cytotoxicity compared to MLE (viable cells 51% vs. 44%, IC_50_ 1.92 ± 0.04 vs. 2.7 ± 0.2 mg/mL, respectively). In conclusion, among the studied plants, MLE has potential for developing as a neuroprotective agent.

## 1. Introduction

Reactive oxygen species (ROS) are a group of oxygen-containing molecules that are highly reactive due to the presence of unpaired electrons; ROS mainly include hydroxyl radical (OH•), superoxide anion (O_2_^−^), and hydrogen peroxide (H_2_O_2_). There are many factors that affect ROS production in cells. The key producer of ROS is environmental stress, and ROS are a common byproduct of the normal metabolic pathway of oxygen molecules. Increased ROS levels either outside or inside the cells result in significant damage to all biological macromolecules, namely lipids, proteins, and nucleic acids. Cells naturally generate ROS such as O_2_^−^ and H_2_O_2_ through one-electron reduction of oxygen to O_2_^−^ catalyzed by an NADPH or NADH oxidase utilizing NADPH or NADH as electron donors. Part of the O_2_^−^ is converted to H_2_O_2_ via spontaneous or enzyme-facilitated dismutase; this reaction is called oxidative burst. The H_2_O_2_ causes significant physiological and pathological effects due to its being highly diffusible and its ability to cross the plasma membrane [1]. Oxidative stress, achieved by the accumulation of ROS, is suggested to be an initiator of neurodegenerative disease.

The brain is an organ that is prone to producing ROS (such as O_2_^−^, H_2_O_2_, and OH•) due to its requiring a large quantity of oxygen to maintain its normal function. Furthermore, neuronal cells are more sensitive to oxidative stress than the cells in other types of tissue. Therefore, the inhibition of oxidative stress is considered to be a strategy to prevent neuronal progressive disease [2]. Oxidative stress leads to apoptotic neuronal cell death by initiating mitochondrial dysfunction, which causes neurodegenerative diseases such as Alzheimer’s disease and Parkinson’s disease; so far, no potent drugs that fully prevent neuronal cell death in neurodegenerative diseases are available [3]. The qualified preservation process of neuronal structure and function is called neuroprotection; its aim is to inhibit or delay disease progression and nerve cell damage by preventing or reducing the loss of neurons [1]. In fact, dietary polyphenols have been shown to display powerful neuroprotective effects as antioxidants. These dietary polyphenols are interesting due to their role in suppressing the oxidation of proteins, lipid peroxidation, and the generation of ROS in diverse in vitro and in vivo models of neurological disorders [4].

Many of the therapeutic strategies that focus on the prevention of the ROS formation mediated by antioxidants seem to have an impact on delaying the disease’s progression. Numerous synthetic antioxidants have been established to be potent radical scavengers, but they are also mutagenic and cause cell damage. Therefore, much attention has recently been paid to antioxidants from natural origins with neuroprotective effects. Various plant species in different regions of the world have been found to be rich sources of several bioactive compounds with potential health benefits. Phenolic compounds, which mainly exist in vegetables, fruits, and dry fruits, are natural antioxidants and are the biggest group of potent antioxidants [5]. Phenolic compounds are classified as “chain breaking antioxidants” due to their ability to chelate transition metal ions, thereby inhibiting oxidative chain reactions in cells [6]. They have been reported to quench free radicals by donating a hydrogen atom and/or an electron to free radicals.

Many plants rich in phenolic compounds are consumed almost daily by Asian people in their diet; selecting and investigating the antioxidant effects of the most commonly used plants will provide a source of natural, reliable, cheap, and safe neuroprotection compounds for everyone. The traditional use of *Moringa oleifera* is reported including for anti-coagulation for snake bites, induction of breast milk production, and hair care products by delivering nutrients to the hair follicles [7]. *Alpinia galanga* is commonly used as a medicinal plant to treat various diseases such as chest pain, rheumatic pains, fever, kidney disease, burning of the liver, and dyspepsia. The seed of *A. galanga* is used for cleaning the mouth, stimulating digestive power, and inducing appetite. Its rhizome is usually used as a spice in Asian communities and consider a rich source of essential oil [8]. In various countries, *Vitis vinifera* are often found in wine, juice, and raisins as well as rootstocks [9]. *Panax ginseng* is popular for aging therapy, psychiatric complications, and remedy disorders of the digestive system [10]. In this study, *M. oleifera* leaves, leaves and rhizomes of *A. galanga*, *V. vinifera* seeds, and leaves and rhizomes of *P. ginseng* were selected for investigation of their antioxidant capabilities and neuroprotective antioxidant activities on a neuroblastoma cell line (SHSY5Y).

SHSY5Y cell line is a subline of the SK-N-SH cell line, established in 1970 from a bone marrow biopsy of a neuroblastoma tumor of a four-year-old female in the metastatic stage that underwent three rounds of clonal selection [11]. In addition, the active compounds contained in *M. oleifera* leaves extract (MLE) related to antioxidant capacity were elucidated by liquid chromatography coupled with quadrupole time-of-flight mass spectrometry (LC–QTOF/MS). This is the first report on the investigation of *M. oleifera* cultivated in Thailand in both antioxidant actions (capacity and activity) as well as providing profile analysis of valuable phenolic compounds, particularly flavonoids. This information could pave the way for further study of their antioxidant activities and effects as a single compound on neuroprotective agents that exhibit significance for mental health protection, particularly those for neurodegenerative disorders induced by oxidative stress.

## 2. Materials and Methods

### 2.1. Plant Materials

Leaves and rhizomes of the selected plant species *A. galanga*, and *P. ginseng* and leaves of *M. oleifera* were obtained from local gardens in Khon Kaen, Thailand in October 2017, whereas *V. vinifera* seeds were provided by Visootha (Nikki) Lohinavy of GranMonte Asoke Valley Winery from a winemaking factory in Pak Chong, Nakorn Rachasima, Thailand. Plant materials were cleaned, dried at 65 °C using a hot air oven (Model FD240, Binder, Frankfurt, Germany), and crushed using a Philips mill (Model 600W, Eindhoven, The Netherlands). The ground materials were kept in screwcap containers at room temperature away from the sun until the beginning of the extraction process.

### 2.2. Preparation of Plant Extracts

All plant samples were subjected to extraction using ethanol with a ratio of 1:4 and stirring with a magnetic stirrer for 8 h, then were filtrated using Whatman No. 1 filter paper (Camlab, Cambridge, UK). *V. vinifera* seeds [12,13], *M. oleifera* leaves [14,15], and *P. ginseng* leaves and rhizomes [16,17] were extracted with 70% ethanol, whereas *A. galanga* leaves and rhizomes were extracted with 95% ethanol [18,19]. Each sample was concentrated using a rotary evaporator (Model Heidolgh VV2000, Heidolph Instruments Gmbh, Schwabach, Germany). The *A. galanga* rhizomes extract (ARE) consisted of two layers, with a pale yellow upper layer (PYL) with oily characteristics and a brown lower layer (BL). *A. galanga* leaves extract (ALE), *P. ginseng* leaves and rhizomes extracts (PLE and PRE), *V. vinifera* seeds extract (VSE) and *M. oleifera* leaves extract (MLE) were obtained in powder form while ARE (both BL and PYL) was in semi-solid form due to the presence of essential oil. Each extract was kept separately in screwcap vials at 4 °C.

### 2.3. Determination of Antioxidant Capacity

#### 2.3.1. Scavenging of the 1,1-Diphenyl-1-Picrylhydrazyl Radical (DPPH)

The estimation of the free DPPH radical system was performed as described in [5]. The reaction was prepared by adding 2.94 mL of 0.1 mM DPPH in methanol into 60 µL of each plant extract, with a final concentration of 200 µg/mL compared to methanol as negative control and gallic acid as positive control (with 95% DPPH scavenging). The reaction mixture was incubated in a dark place for 30 min, then vigorously shaken for 20 s. The decrease in absorbance at 516 nm was recorded using a UV Probe-1800 spectrophotometer (Shimadzu, Kyoto, Japan). The percentage of DPPH radical scavenging was calculated using the following equation:

% DPPH scavenging = [(*Ab*_0_ − *Ab*_1_/*Ab*_0_)] × 100 (1)

where *Ab*_0_ is the absorbance of the control (no extract) and *Ab*_1_ is the absorbance of the test extract.

#### 2.3.2. Ferric-Reducing Antioxidant Power (FRAP)

The FRAP assay was achieved according to [20] with some modifications. The FRAP solution was prepared by mixing 25 mL of 300 mM acetate buffer, pH 3, with 2.5 mL of 10 mM TPTZ (2,4,6-tripyridyl-s-triazine) in 40 mM HCl and 2.5 mL of 20 mM of Iron(III) chloride hexahydrate (FeCl_3_. 6H_2_O). Distilled water was used as a negative control while Trolox was used as a positive control (with 0.03 ± 0.1 mg/mL for ferric-reducing power). The reaction was started by adding 100 µL of crude extract into 2 mL of the FRAP working solution and kept for 30 min in dark conditions. The absorption of the ferrous tripyridyltriazine complex was recorded at 593 nm. The standard curve was obtained by various concentrations of ferrous sulfate ranging from 5 to 100 µM/mL. The results of FRAP were expressed in μmol Fe (ΙΙ)/g dry weight.

### 2.4. Neuroblastoma Cell Cultures (SHSY5Y)

The SHSY5Y cell line (human neuroblastoma) (The American Type Culture Collection (ATCC^®^), CRL2266™, Manassas, VA, USA) was obtained from the Institute of Biotechnology and Genetic Engineering, Chulalongkorn University, Bangkok, Thailand. The medium DMEM/F-12 containing Dulbecco’s modified Eagle’s medium (DMEM) and Ham’s F-12 Nutrient Mixture at a ratio of 1:1 was used in the SHSY5Y culture. Cells were grown in DMEM/F-12 medium containing 1 mM sodium pyruvate, 0.1 mM nonessential amino acid, 1.5 g/L sodium bicarbonate, 100 U/mL penicillin, and 100 µg/mL streptomycin supplemented with 10% heat-activated fetal bovine serum (FBS) (Thermo Fisher Scientific, Waltham, MA, USA) and incubated at 37 °C in an incubator with 85% humidified atmosphere containing 5% CO_2_.

#### 2.4.1. Culturing and Harvesting of the SHSY5Y Cells

SHSY5Y cells in a culture flask were trypsinized and re-suspended in the medium, and 100 µL of 2 × 10^4^ cells/well were plated in 96-well culture plates. After 24 h, cells were used for further investigations.

#### 2.4.2. Cytotoxicity of Plant Extracts on the SHSY5Y Cell Line

The cellular viability assay described in [21,22] was used to investigate cytotoxicity on the SHSY5Y cell line. Incubated cells as described in the previous step were exposed to 100 µL of crude extracts with concentrations ranging from 0.25 to 4 mg/mL. The negative control did not contain an extract. Cells were incubated in 5% CO_2_ atmosphere at 37 °C for 24 h. Then, the medium was removed, 100 µL of 0.5 mg/mL 3-(4,5-dimethylthiazol-2-yl)-2,5-diphenyltetrazolium bromide (MTT) in Dulbecco’s phosphate buffer saline (DPBS) was added, and cells were incubated for an additional 4 h. Finally, MTT solution was gently removed and 100 µL of dimethyl sulfoxide (DMSO) was added to dissolve crystal formazan. Triton X-100 was used as positive control (considered as 100% cell death). The plate was shaken for 1 min, and the absorbance of formazan in each well was measured in a microplate reader at 570 nm. The percentage of cytotoxicity compared with the negative control (untreated cells, considered as 100% cells viability) was calculated according to the following equation:

Cytotoxicity % = (Ab. of control cells − Ab. of treated cells)/(Ab. of control cells) × 100(2)

The plot of percent cytotoxicity versus sample concentration was used to calculate the extract concentration that killed 50% of the cells (IC_50_).

#### 2.4.3. Neuroprotection of Plant Extracts on the SHSY5Y Cell Line

H_2_O_2_ was used to induce oxidative stress in this study. SHSY5Y cells were treated with H_2_O_2_ in the range of 100–500 μM and incubated for 24 h, and 100 μL of 250 μM H_2_O_2_, which inhibited 70%–80% cell viability, was selected to examine the neuroprotective effects of plant extracts. To assess the dose-dependent neuroprotective effects of the plant extracts against oxidative-stress-induced neuron cells, cells were pretreated with each tested plant extract (IC_50_ was selected based on MTT assay) for 6 h and then exposed to 250 μM H_2_O_2_ for 1 h. The cytotoxic effect was calculated as a percentage of viability when compared with untreated cells (negative control considered as 100% of viability) and cells treated with 250 μM H_2_O_2_ for 1 h (positive control considered as 100% of cell death) [4]. After each assay, cell viability was measured using the MTT assay as described in Section 2.4.2.

### 2.5. Determination of Intracellular O_2_^−^ and ROS inside the SHSY5Y Cell Line

#### 2.5.1. Determination of O_2_^−^ by Nitro-Blue Tetrazolium (NBT) Reduction Test

The reduction of NBT to insoluble blue formazan was used as a probe for O_2_^−^ generation inside the viable cells according to the method in [23] with slight modification. Supernatants of SHSY5Y cells described in Section 2.4.1 were eliminated and replaced by a medium with different concentrations (0.25, 0.5, 1, 2, and 4 mg/mL) of tested plant extracts, then 15 µL of 1% NBT solution was added. Untreated cells (negative control: considered as moderate absorbance) and cells treated with 200 μM H_2_O_2_ for 1 h (positive control: considered as massive absorbance). Following 3 h incubation, the supernatants were removed, and the formation of formazan product was solubilized in 100 µL DMSO. After mixing the contents in each well, the absorption of colored formazan solution was read at 620 nm using a Varioskan LUX multimode microplate reader (Thermo Fisher Scientific, Waltham, MA, USA).

#### 2.5.2. Determination of Intracellular ROS by DCFHDA Assay

To investigate the formation of ROS inside SHSY5Y cell line with and without plant extracts, 2,7-dichlorodihydrofluorescein diacetate (DCFHDA) was used [24]. Briefly, SHSY5Y cells were incubated with various concentrations of plant extract (0.25, 0.5, 1, 2, and 4 mg/mL) to induce the antioxidant system in cells using bioactive compounds. After 3 h of incubation, cells were washed with Dulbecco’s phosphate buffer saline (DPBS) and then incubated for 60 min in 10 μM DCFHDA (Molecular Probes, Invitrogen, Basel, Switzerland) in complete medium at 37 °C and 5% CO_2_. After the washing step with DPBS, the cells were treated with 200 µM of H_2_O_2_ for 2 h to undergo intracellular oxidation. Untreated cells were negative control (considered as moderate absorbance) and cells treated with 200 μM H_2_O_2_ for 1 h were positive control (considered as massive absorbance). Fluorescence intensity was measured using the Varioskan LUX multimode microplate reader (Thermo Fisher Scientific, Waltham, MA, USA) at an excitation wavelength of 485 nm and an emission wavelength of 528 nm.

SHSY5Y cells were incubated separately with different volumes of DMSO (solvent used for solubilizing plant extracts) to check the viability of cells.

### 2.6. Liquid Chromatography–Quadrupole Time-of-Flight Mass Spectrometer (LC–QTOF/MS) Analysis

A MLE sample with a concentration of 101.23 mg/mL in 50% methanol was prepared, ultrasonicated for 20 min, and centrifuged at 10,000 rpm at 4 °C for 10 min. The supernatant was filtrated with 0.2 µm nylon membrane and injected into liquid chromatography coupled with a quadrupole time-of-flight mass spectrometer (LC–QTOF/MS) (1290 Infinity II LC-6545 Quadrupole-TOF, Agilent Technologies, Santa Carla, CA, USA). The liquid chromatographic system consisted of a binary pump and an online vacuum degasser connected to a Dual AJS ESI source mass spectrometer (Agilent Technologies, Santa Carla, CA, USA). Full-scan mode was used from m/z 100 to 1700. Zorbax Eclipse Plus column (Agilent Technologies, Santa Carla, CA, USA) (C18 2.1 × 150 mm, 1.8 µm) was used for the analysis. Formic acid 0.1% in distilled water (solvent A) and 100% acetonitrile (solvent B) were used as the mobile phase-gradient elution as follows: 98% A, 0–2 min; 90% A, 2–25 min; 85% A, 25–40 min; 80% A, 40–48 min; 75% A, 48–68 min; 70% A, 68–80 min; 50% A, 80–85 min; 0% A, 85–90 min; 98% A, 90–100 min. Peaks were detected at wavelengths of 254 and 280 nm. The MS spectra were acquired in both positive and negative ion modes, auto MS/MS. The mass fragmentations were identified using the spectrum database for organic compounds in the METLIN database (a cost-free reachable web-based data source, has been developed to facilitate in a wide range of metabolite research and to assist natural products identification through mass analysis).

### 2.7. Statistical Analysis

All investigations were conducted in this study in triplicate (*n* = 3). Data were reported as mean ± SD values. Analysis of variant (ANOVA) was used to evaluate the differences among the treatments by Duncan’s multiple range tests (DMRTs) with *p* value of 0.05 using SPSS Statistics Base version 19 program for Windows (IBM Corp, Chicago, IL, USA). 

## 3. Results and Discussion

In the present study, the antioxidant capacities and neuroprotective activities of the four selected Asian plants—*M. oleifera*, *A. galanga*, *V. vinifera*, and *P. ginseng*—were investigated. Different parts of these plants were extracted with 70% and 95% ethanol, which is a polar solvent. Therefore, the compounds in the plant extracts were those with high polarity. The extracts were assayed for their antioxidant capacity by FRAP and DPPH methods. The DPPH assay is achieved based on both hydrogen atom transfer (HAT) and single electron transfer (SET) mechanisms [25]. VSE gave the highest activity in quenching DPPH radicals, followed by MLE, with 81% and 58%, respectively (Table 1). The ability of VSE and MLE extracted with 70% ethanolic solvent in scavenging DPPH radicals agreed with the results obtained by [26,27,28], who reported that catechin and epicatechin are the most abundant compounds among phenolic components in VSE. Luteolin seems to play a role in the antioxidant capacity of MLE [29]. Table 1 also shows that ALE gave the highest activity of FRAP, followed by MLE (33.57 and 26.76 μmol Fe (ΙΙ)/g dry wt., respectively). VSE showed moderate ferric reducing activity, with 19.45 μmol Fe (ΙΙ)/g dry wt. PRE, PLE, and ARE (PYL) had low FRAP activity, which implied that these have the lowest electron donation ability. The FRAP activity of ALE may be explained by the presence of the galango flavonoid, which was reported in ethanolic extract of the galanga plant. Previous study reported that galango flavonoids are effective in free radicals scavenging as well as metal chelating activity [30]. Meanwhile, dried MLE provide a rich source of polyphenolic compounds such as phenolic acids and flavonoids; both subgroups are characterized as highly effective antioxidant molecules [31]. Although the ranking of the tested extracts’ capacities varied in both (DPPH and FRAP) tests, MLE holds on as the second most active performer.

The data are expressed as mean ± SD (*n* = 3) while for DPPH and neuroprotection results expressed in cell viability percentage, (μmol/g): μmol of Fe (ΙΙ)/g of dry weight plant extract). ND (not determined): extract excluded from viable system (SHSY5Y) experiments due to high cytotoxicity. a, b, c, d, e, f, g are significant differences with *p*-value (<0.05). MLE: *M. oleifera* leaves extract; VSE: *V. Vinifera* seeds extract; PLE: *P. ginseng* leaves extract; PRE: *P. ginseng* rhizomes extract; ALE: *A. galanga* leaves extract; ARE (PYL): *A. galanga* rhizomes extract (pale yellow layer); ARE (BL): *A. galanga* rhizomes extract (brown layer).

According to its high antioxidant capacity in scavenging DPPH radicals and FRAP activity, MLE was selected for further investigation of its phytochemical contents by LC–QTOF/MS analysis. The results in Table 2 reveal that MLE contained barbatoflavan, isorhamnetin, quercetin derivatives, kaempferol derivative, caffeoylquinic acid, coumaroyltrifolin, naringenin, pheophytin, and vitexin. These compounds have the ability to scavenge DPPH and have reducing power (FRAP) by improving hydrogen-atom-transfer (HAT) and electron-donation-based pathways.

All of the studied plants are well known in the daily life of Asian people. After comparison of their properties, the results showed that MLE had better biological activities than any others. It showed the unique antioxidant characteristic inside and outside viable systems. As the main objective of this study was screening the most powerful plant used in the daily Asian diet, MLE was therefore selected to analyze its composition. 

It is known that antioxidant compounds in plants have benefits for the prevention or treatment of various disease conditions, including neurodegenerative diseases. Moreover, polyphenolic compounds possess numerous biological activities such as anti-inflammation and enzyme inhibition activities, gene expression, and signal transductions that play a significant role in health. In the present study, the evaluation of neuroprotective effect achieved on the human neuroblastoma (SHSY5Y) cell line was performed. This neuronal cell line was selected for the following reasons: (a) this cell line is acquired from a human origin, a source from which primary neurons are difficult to obtain (b) cells have been proposed as a useful model for studying the effect of free radicals formation and scavenging in the human neurons as they possess many biochemical and functional properties of neurons (c) the cell population is reasonably homogenous, which hypothetically translates into reproducibility and (d) these cells are oncogenic and their proliferation to yield a sufficient quantity for investigations can be accomplished.

Before investigation of their neuroprotection activities, all of the studied plant extracts were tested for cytotoxicity on SHSY5Y cells by MTT assay. The results of 50% inhibition concentration (IC_50_) of the extracts are summarized in Table 1. MLE, ALE, and VSE showed the lowest cytotoxicity, with IC_50_ values of 2.7 ± 0.2, 2.53 ± 0.1, and 2.43 ± 0.1 mg/mL, respectively, whereas ARE (PYL) showed the most cytotoxic effect to SHSY5Y cells with an IC_50_ of 0.34 ± 0.02 mg/mL. The data of ARE (BL) showed high toxicity on the neuroblastoma cell line, even in low concentrations. After testing the cytotoxicity of each extract and optimizing the lethal concentration of H_2_O_2_ on SHSY5Y cell line, the extracts were investigated for their neuroprotection activity in the presence of H_2_O_2_. It was found that PRE provided the greatest amount of neuroprotection, with 51% of viable cells, followed by MLE and ARE (PYL), with 44% and 40% of viable cells, respectively. Several studies reported that ginsenosides in PRE can support brain function, prevent neuroinflammation and oxidative stress, and inhibit or weaken various neurodegenerative disorders such as Alzheimer’s disease, Huntington’s disease (HD), and traumatic brain injury [32]. MLE can improve the memory of Alzheimer’s disease patients by significantly increasing superoxide dismutase (SOD) and catalase which are the antioxidant enzymes, and also by reducing lipid peroxidase levels. These antioxidant activities may improve cognitive functions [33]. ARE acts as a suppressor of lipid peroxidation in the brain by preserving the activities of endogenous antioxidant enzymes, including SOD, catalase, and other antioxidant molecules such as glutathione, which could reduce oxidative stress. Aqueous and alcoholic extract of ARE was shown to be a powerful scavenger of ROS that encouraged the progression of neurodegenerative disorders [34].

**Figure 1:**
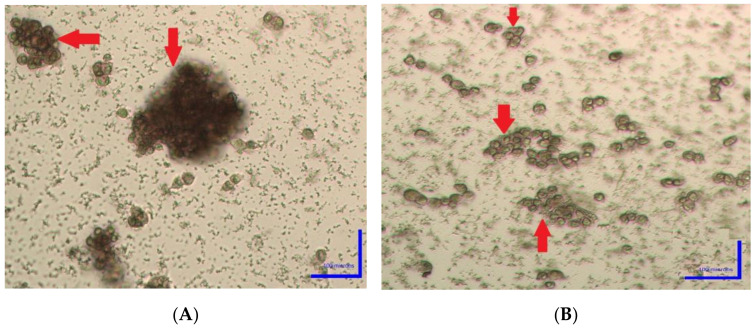
Nitro-blue tetrazolium (NBT) reduction test: (**A**) control cells (SHSY5Y cells without MLE); (**B**) SHSY5Y cells treated with MLE.

Using the NBT test, we assayed different plant extracts for their capacity to protect the SHSY5Y cell line from O_2_^−^ radicals; the results are summarized in Table 1. MLE showed the best NBT reduction with the lowest IC_50_ concentration (0.83 ± 0.09 mg/mL), followed by VSE (0.98 ± 0.08 mg/mL). ALE and PRE showed less reduction activity—1.24 ± 0.05 and 1.22 ± 0.06 mg/mL, respectively. ARE (PYL) and PLE showed the lowest O_2_^−^ radical scavenging activities—1.62 ± 0.1 and 1.65 ± 0.04 mg/mL, respectively. Figure 1A,B show the difference between control cells; SHSY5Y cells without MLE (dark colored with high O_2_^−^) and treated cells with MLE (light colored with low O_2_^−^).

**Figure 2:**
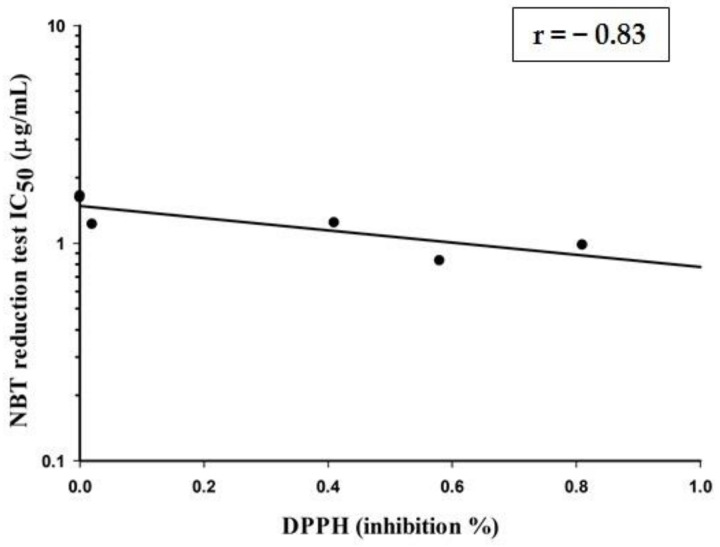
The correlation between the NBT reduction test and DPPH inhibition activity of plant extracts (*r* = − 0.83, *p* < 0.05).

Correlations between the free radical scavenging effect of tested extracts (MLE, VSE, PLE, PRE, ARE (PYL) and ALE) outside the cells by DPPH and FRAP tests and inside the viable cells by scavenging O_2_^−^ and intracellular ROS were determined using Pearson coefficient correlation. The strongest and best correlation was found between DPPH and IC_50_ of NBT reduction activity with a value of *r* = −0.83, *p* < 0.05; the results of the correlation established that extracts that possess higher quenching DPPH values are able to scavenge O_2_^−^ with lower IC_50_ in the NBT test, as shown in Figure 2. The other correlations showed non-significant correlation (*p*-value > 0.05).

To detect the capacity of the obtained plant extracts to scavenge intracellular levels of ROS in SHSY5Y cells, DCFHDA was applied as a fluorogenic substrate. This substrate was converted to highly fluorescent DCFH by ROS and was monitored by the spectrophotometer. Table 1 shows the concentrations of extracts that inhibit 50% of intracellular ROS levels by the DCFHDA assay. MLE, PRE, and ARE (PYL), with IC_50_ values of 0.71 ± 0.08, 0.83 ± 0.08, and 0.96 ± 0.1 mg/mL, respectively, exhibited the best ROS scavenging activity. VSE (1.24 ± 0.06 mg/mL) and PLE (1.34 ± 0.06 mg/mL) showed less effectiveness in decreasing intracellular ROS levels, whereas ALE (2.48 ± 0.03 mg/mL) showed the lowest antioxidant activity in SHSY5Y cells. Moreover, SHSY5Y cells treated with DMSO showed no effect on the cells. The unique activity of MLE might be due to the presence of potent compounds that are commonly known to have antioxidant efficiency, such as quercetin derivatives, isorhamnetin glucoside, kaempferol, luteolin and its derivatives, ascorbic acid derivative, diosmetin, apigenin and its derivatives (saponarin), hydroxytyrosol 1-*O*-glucoside (HT), and esculetin, as shown in Table 2. Ginsenoside, the main active ingredient in PRE, could protect SHSY5Y from reactive oxygen species by the Bcl-2-associated X protein (BAX) pathway [35]. Coumarins, sesqiterpenes, and volatile oil, including eugenol and its derivatives in ARE, could protect ROS in 4T1 breast cancer cells and NIH-3T3 fibroblast cells [36].

From the data mentioned above, this extract ranked as first or second place in all experiments; the revealed data established that ethanolic MLE extract is pharmacologically more active than other tested extracts, which might be because of the synergistic effects of various potent components present in the whole extract.

**Figure 3:**
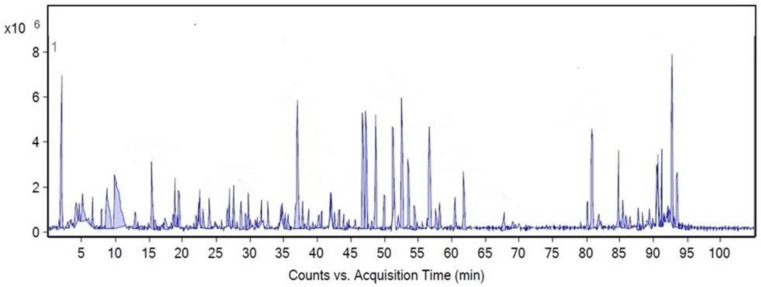
Liquid chromatography coupled with quadrupole time-of-flight mass spectrometry (LC–QTOF/MS) chromatogram of MLE.

Taken together, MLE, grown in Thailand, is interesting as a potential neuroprotective agent with further applications due to its low cytotoxicity and high antioxidant activities. LC–QTOF/MS analysis (Figure 3 and Table 2) of MLE in the present study, confirmed by the fragmentation MS spectra (see the Supplementary Materials Figure S1), showed that the extract was significantly rich in antioxidant ingredients (31 compounds) compared with constituents found in moringa leaves cultivated in China and India (11 compounds that were also present in our extract) [37], whereas the phytochemicals reported in a moringa extract from Malaysia include multiflorin-B and derivatives of apigenin, quercetin, and kaempferol [38]. This means that the moringa plant cultivated in Thailand ranks as one of the best sources of potent antioxidant compounds. The difference in phytochemical constituents might be due to the plants’ adaptation to the climatic environment and soil factors such as soil type, pH, and soil nutrients [39]. Therefore, concentrations of these constituents are usually associated with factors such as the environment and growing areas [40]. In Table 2, MLE is shown to contain eriodictyol and gallic acid, which play a role in scavenging H_2_O_2_ and preventing cell damage by oxidative stress. It also contained various effective phenolic compounds that support a powerful antioxidant activity, including apigenin 7-rhamnosyl-(1→2)-galacturonide, cartormin, kaempferol 4′-glucoside, hesperetin, and isorhamnetin 3-(6″-acetylglucoside).

MLE requires more attention, as it plays an important and unique biological role as an ecofriendly and non-cytotoxic antioxidant agent compared to other tested extracts in all tests.

**Table 2:** List of compounds identified in MLE by LC–QTOF/MS analysis.

Compound	Molecular Formula	Retention Time (min)	Candidate Mass	Effect	Reference
Barbatoflavan	C_24_H_28_O_13_	33.051	525.15	-Established scavenging properties toward the DPPH radicals	[41]
Isorhamnetin	C_16_H_12_O_7_	83.499	315.05	-Scavenging DPPH free radical	[42]
Quercetin	C_15_H_10_O_7_	48.551	303.04	-Scavenging O_2_^−^ and DPPH radicals	[43]
Quercetin 3-(6″-malonylglucoside)-7-rhamnoside	C_30_H_32_O_19_	39.686	697.16	-Scavenging O_2_^−^ and DPPH radicals	[43]
Quercetin 3-*O*-(6-*O*-malonyl-β-d-glucoside)	C_24_H_22_O_15_	51.250	549.08	-Scavenging O_2_^−^ and DPPH radicals	[43]
Quercetin 3-methyl ether	C_16_H_12_O_7_	53.451	317.06	-Free radical scavenging -Inhibitory effect on O_2_^−^ generation	[44,45]
Kaempferol 3-[6‴-*p*-coumarylglucosyl-(1→2)-rhamnoside]	C_36_H_36_O_17_	61.466	741.20	-Improves the potency of the hydrogen-atom-transfer (HAT)-based pathways	[46]
4,5-Di-*O*-caffeoylquinic acid	C_25_H_24_O_12_	69.978	515.11	-Ability to protect cells by conducting electron transfer (ET), H^+^ transfer, and Fe^2+^ chelation	[47]
6″-*O*-*p*-Coumaroyltrifolin	C_30_H_26_O_13_	72.336	593.12	-Improving the ET- and HAT-based pathways -Boosts Fe^2+^ -Chelating ability	[48]
(±)-Naringenin	C_15_H_12_O_5_	78.072	271.06	-Electron-donating substituents via weakening the bond dissociation enthalpy (BDE)	[49,50]
Pheophytin	C_55_H_74_N_4_O_5_	92.966	869.55	-Fe(II) chelator -Suppresses lipid peroxidation -The conjugated double bonds presented in the porphyrin ring can act as electron transfer that will stabilize a radical compound	[51,52]
Vitexin 4′-*O*-galactoside	C_27_H_30_O_15_	40.024	593.15	-Electron donor that may act as a good radical scavenger -Suppresses O_2_^−^ generation by promoting superoxide dismutase	[53]
Luteolin 7-methyl glucuronide	C_22_H_20_O_12_	41.903	475.09	-Suppresses O_2_^−^ generation	[54]
Quercetin 7-(6″-acetylglucoside)	C_23_H_22_O_13_	51.213	515.10	-Influence on O_2_^−^ -Reduction in radicals -Decreases intracellular hydrogen peroxide accumulation	[55]
Isorhamnetin 3-(6″-acetylglucoside)	C_24_H_24_O_13_	58.203	519.11	-Free radical scavenger -Inhibits O_2_^−^ production -Suppresses lipid peroxidation by preventing the conversion of hydrogen peroxide into hydroxyl radical by the Haber–Weiss reaction	[55]
Saponarin (apigenin-6-C-glucosyl-7-*O*-glucoside)	C_27_H_30_O_15_	37.047	593.14	-O_2_^−^ scavenging activity -Neutralizes hazardous free radicals	[56]
Apigenin	C_15_H_10_O_5_	79.602	269.04	-Scavenges O_2_^−^ by reversed decreasing of superoxide dismutase	[57]
Quercetin 3-galactoside	C_21_H_20_O_12_	48.637	713.15	-Scavenges ROS	[58]
Kaempferol	C_15_H_10_O_6_	35.262	287.05	-Efficiently prevents ROS generation	[59]
Luteolin	C_15_H_10_O_6_	80.827	285.04	-Scavenges O_2_^−^ -Inhibits H_2_O_2_ production and scavenges H_2_O_2_ -Reduction in ROS production	[60]
Luteolin 7-(6′′′-acetyl allosyl-(1→2)-glucoside)	C_29_H_32_O_17_	37.044	651.16	-Ability to scavenge ROS	[26]
l-Ascorbic acid-2-glucoside (AA2G)	C_12_H_18_O_11_	3.129	337.08	-Directly interacts and scavenges free radicals -Suppresses H_2_O_2_, which induces oxidative stress	[61]
Diosmetin	C_16_H_12_O_6_	63.793	299.05	-Slight decrease in ROS	[62]
Esculetin	C_9_H_6_O_4_	21.757	177.01	-Attenuates ROS production to approximately 40%	[63]
Apigenin 7-rhamnosyl-(1→2)-galacturonide	C_27_H_28_O_15_	56.514	591.13	-Supports a powerful antioxidant activity	[64]
Cartormin	C_27_H_29_NO_13_	45.007	574.15	-Increases the total antioxidative activity	[65]
Kaempferol 4′-glucoside	C_21_H_20_O_11_	52.776	447.09	-Supports a powerful antioxidant activity	[66]
Eriodictyol	C_15_H_12_O_6_	54.307	259.06	-Protects cells against oxidative-stress-induced cell damage	[67]
Hesperetin	C_16_H_14_O_6_	79.820	301.07	-Supports antioxidant activity	[49]
Hydroxy tyrosol 1-*O*-glucoside (HT)	C_14_H_20_O_8_	13.423	315.10	-Scavenges the free radicals -Scavenges O_2_^−^ by promoting superoxide dismutase activity	[68]
Gallic acid	C_7_H_6_O_5_	49.582	169.01	-Protects cells from free-radical-induced cell damage	[69]

## 4. Conclusions

A variety of phytochemicals have been reported to prevent the risk of numerous diseases, including neurodegenerative diseases. In Asian countries, many researchers are seeking bioactive compounds from edible plants or herbs for the prevention or treatment of neurodegenerative diseases. *A. galanga*, *V. vinifera*, *M. oleifera*, and *P. ginseng* cultivated in Thailand were selected in the present study.

MLE showed high antioxidant activities (DPPH and FRAP assays) with low cytotoxicity to SHSY5Y cell lines compared to the other studied plants. Its mechanism of action for neuroprotective effect on SHSY5Y cells is probably due to its high level of polyphenolic and other antioxidant compounds, and it possesses the ability to scavenge free radicals or activate the cellular antioxidant system. LC–QTOF/MS analysis confirmed that MLE consists of phenolic compounds, which are a good source of antioxidants. The determination of pure bioactive compounds involved in neurodegeneration contained in MLE should be the subject of further investigation.

## Figures and Tables

**Table 1:** Cellular viability and antioxidant capacity and activity of selected plant extracts estimated by various tests (1,1-diphenyl-1-picryl hydrazyl (DPPH), ferric-reducing antioxidant power (FRAP), nitro-blue tetrazolium (NBT), intracellular reactive oxygen species (ROS) level by 2,7-dichlorodihydrofluorescein diacetate (DCFHDA), and neuroprotection activity).

Plant Extract	DPPH (Inhibition %)	FRAP (μmol/g)	Cytotoxicity by MTT Assay (IC_50_ mg/mL)	NBT Reduction Test (IC_50_ mg/mL)	Intracellular ROS Levels by DCFHDA (IC_50_ mg/mL)	Neuroprotection (% Cell Viability)
MLE	58	26.76 ± 0.3 ^e^	2.7 ± 0.2 ^a^	0.83 ± 0.09 ^c^	0.71 ± 0.08 ^c^	44
VSE	81	9.45 ± 0.08 ^d^	2.43 ± 0.1 ^b^	0.98 ± 0.08 ^c^	1.24 ± 0.06 ^a^	38
PLE	0.05	7.46 ± 0.2 ^a^	1.96 ± 0.02 ^c^	1.65 ± 0.04 ^a^	1.34 ± 0.06 ^a^	34
PRE	2	10.05 ± 0.08 ^c^	1.92 ± 0.04 ^c^	1.22 ± 0.06 ^b^	0.83 ± 0.08 ^c^	51
ALE	41	33.57 ± 0.2 ^g^	2.53 ± 0.1 ^b^	1.24 ± 0.05 ^b^	2.48 ± 0.03 ^d^	32
ARE (PYL)	0.01	5.62 ± 0.02 ^b^	0.34 ± 0.02 ^d^	1.62 ± 0.1 ^a^	0.96 ± 0.1 ^b^	40
ARE (BL)	31	24.19 ± 0.1 ^f^	ND	ND	ND	ND

## Data Availability

Not applicable.

## Supplementary Materials

The following are available online at https://www.mdpi.com/article/10.3390/plants10050889/s1, Figure S1: Mass spectrum of antioxidant components of MLE.
