# Supplementary material for: Neuroprotective Assessment of Moringa oleifera Leaves Extract against Oxidative-Stress-Induced Cytotoxicity in SHSY5Y Neuroblastoma Cells
Source: Plants (Basel). 2021 Apr 28;10(5):889. doi: 10.3390/plants10050889 (PMC8146478; doi:10.3390/plants10050889)
Supplement: Supplementary file 1 [file plants-10-00889-s001.zip › plants-1145710-supplementary.pdf]

Supplementary Material

Neuroprotective Assessment of Moringa oleifera Leaves Extract against Oxidative-Stress-Induced Cytotoxicity in SHSY5Y Neuroblastoma Cells

Farah Jabbar Hashim [1,2,3], Sukanda Vichitphan [2,4], Patcharee Boonsiri [5] and Kanit Vichitphan [2,4,*]

Figure S1. Mass spectrum of antioxidant components of MLE, (1) Barbatoflavan; (2) Isorhamnetin; (3) Quercetin; (4) Quercetin 3-(6″-malonylglucoside)-7-rhamnoside; (5) Quercetin 3-O-(6-O-malonyl-β-D-glucoside); (6) Quercetin 3-methyl ether; (7) Kaempferol 3-[6‴-p-coumarylglucosyl-(1->2)-rhamnoside]; (8) 4,5-Di-O-caffeoylquinic acid; (9) 6″-O-p-Coumaroyltrifolin; (10) (±)-Naringenin; (11) Pheophytin; (12) Vitexin 4′-O-galactoside; (13) Luteolin 7-methyl glucuronide; (14) Quercetin 7-(6″-acetylglucoside); (15) Isorhamnetin 3-(6″-acetylglucoside); (16) Saponarin (apigenin-6-C-glucosyl-7-O-glucoside); (17) Apigenin; (18) Quercetin 3-galactoside; (19) Kaempferol; (20) Luteolin; (21) Luteolin 7-(6‴-acetyl allosyl-(1->2)-glucoside); (22) L-Ascorbic acid-2-glucoside (AA2G); (23) Diosmetin; (24) Esculetin; (25) Apigenin 7-rhamnosyl-(1->2)-galacturonide; (26) Cartormin; (27) Kaempferol 4′-glucoside; (28) Eriodictyol; (29) Hesperetin; (30) Hydroxy tyrosol 1-O-glucoside (HT); (31) Gallic acid

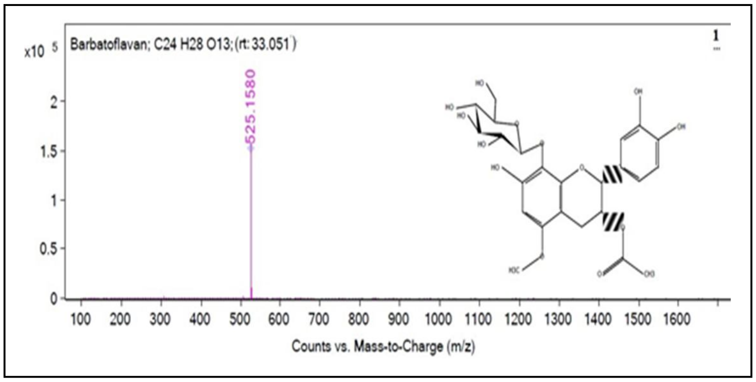

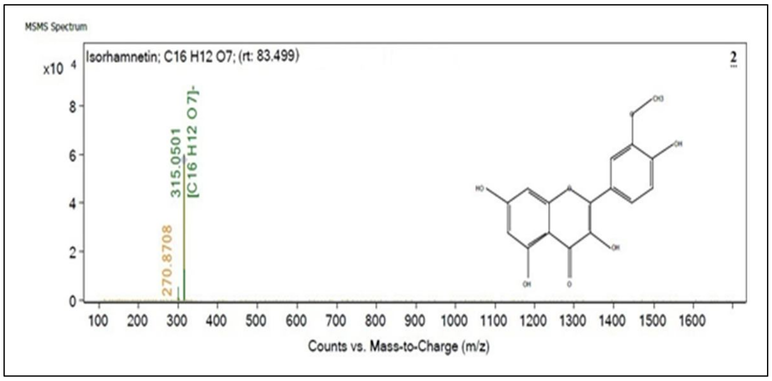

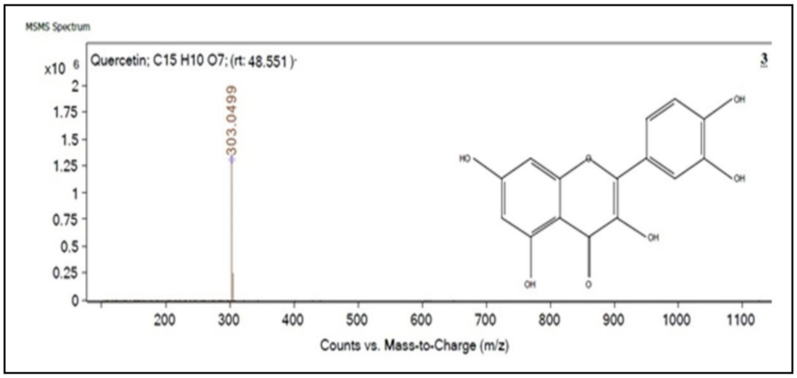

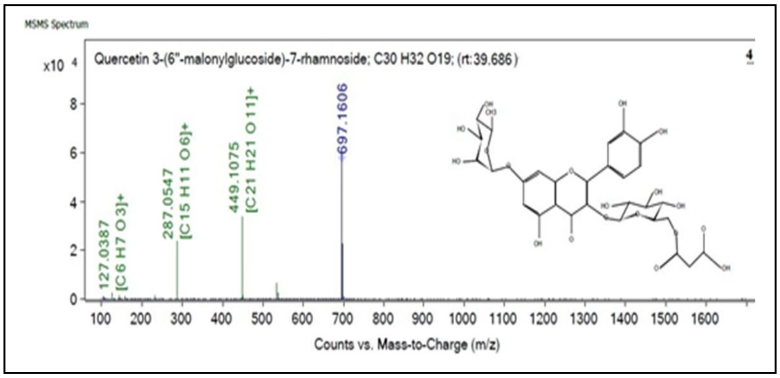

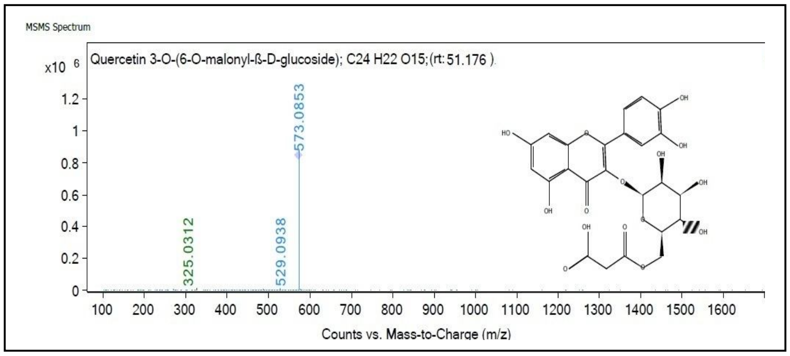

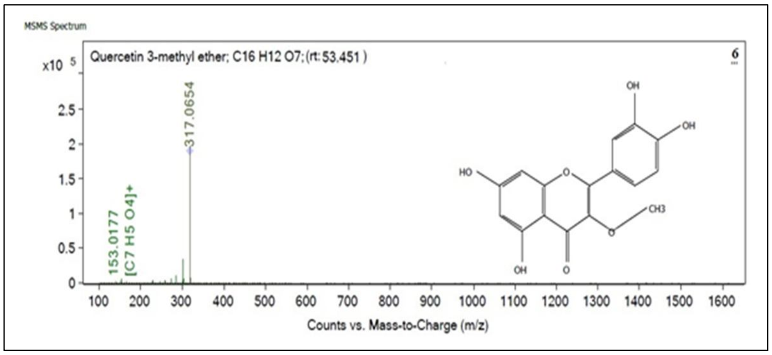

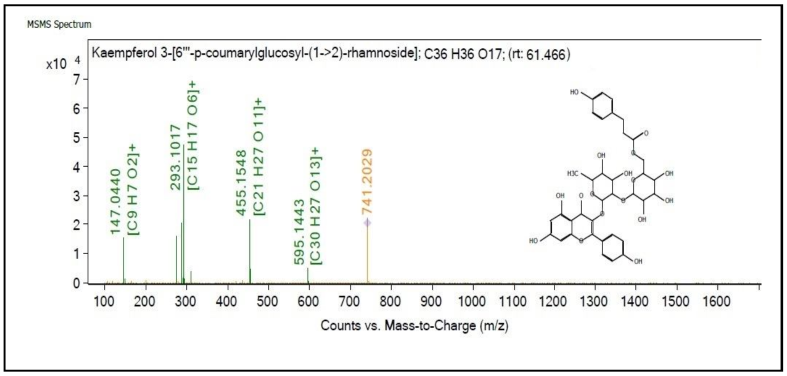

MSMS Spectrum
Kaempferol 3-[6'''-p-coumarylglucosyl-(1->2)-rhamnoside]; C36 H36 O17; (rt: 61.466)
x10 4
147.0440 [C9 H7 O2]+
293.1017 [C15 H17 O6]+
455.1548 [C21 H27 O11]+
595.1443 [C30 H27 O13]+
741.2029
Counts vs. Mass-to-Charge (m/z)

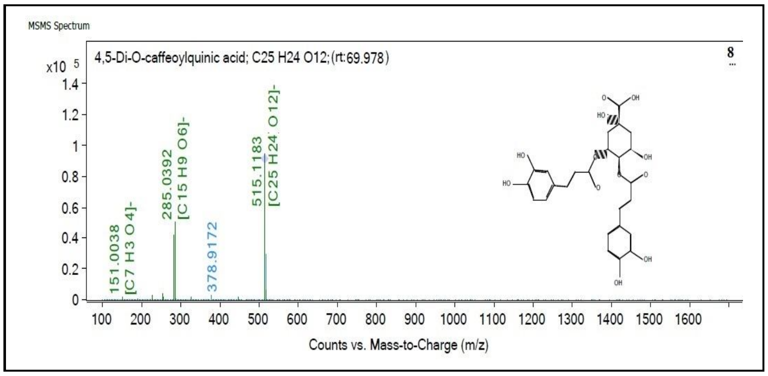

MSMS Spectrum
4,5-Di-O-caffeoylquinic acid; C25 H24 O12; (rt:69.978)
8
x10 5
151.0038 [C7 H3 O4]-
285.0392 [C15 H9 O6]-
378.9172
515.1183 [C25 H24 O12]-
Counts vs. Mass-to-Charge (m/z)

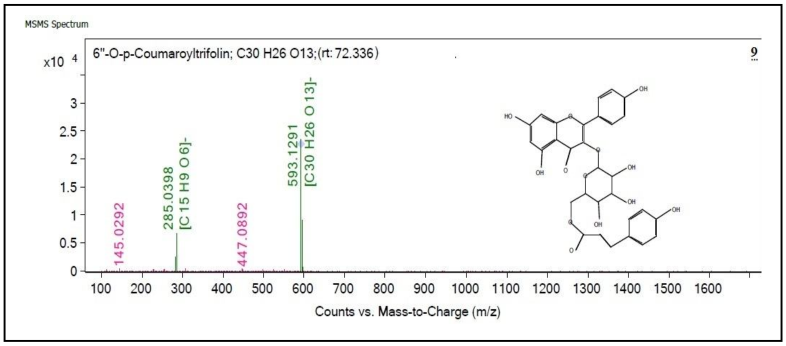

MSMS Spectrum
6''-O-p-Coumaroyltrifolin; C30 H26 O13; (rt: 72.336)
9
x10 4
145.0292
285.0398 [C15 H9 O6]-
447.0892
593.1291 [C30 H26 O13]-
Counts vs. Mass-to-Charge (m/z)

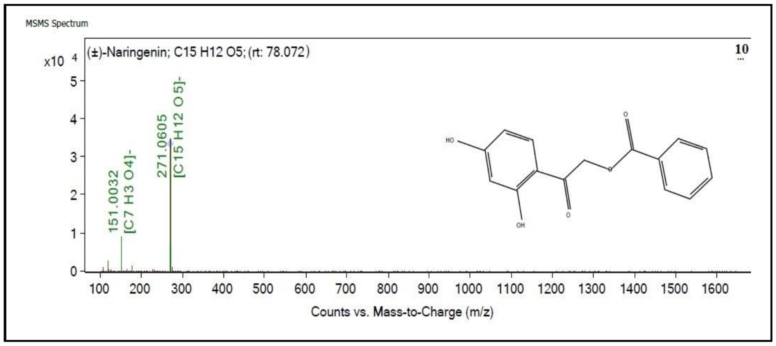

MSMS Spectrum
(±)-Naringenin; C15 H12 O5; (rt: 78.072)
10
x10 4
151.0032 [C7 H3 O4]-
271.0605 [C15 H12 O5]-
Counts vs. Mass-to-Charge (m/z)

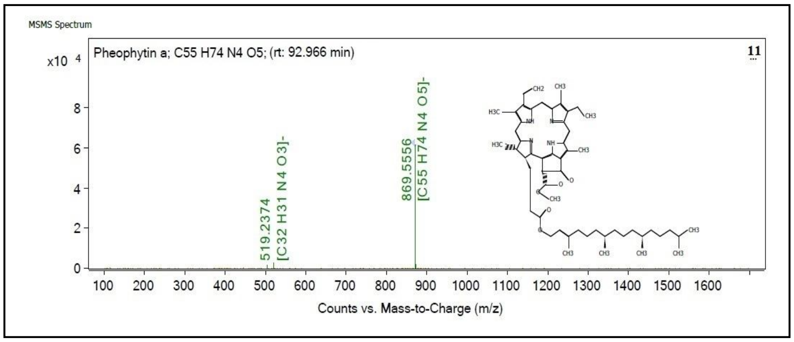

MSMS Spectrum
Pheophytin a; C55 H74 N4 O5; (rt: 92.966 min)
11
x10 4
519.2374 [C32 H31 N4 O3]-
869.5556 [C55 H74 N4 O5]-
Counts vs. Mass-to-Charge (m/z)

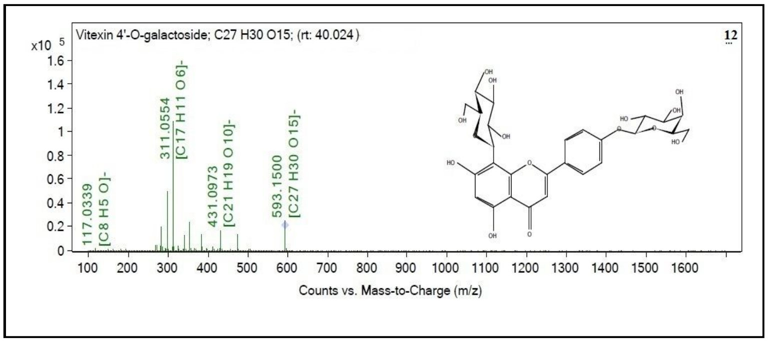

Vitexin 4'-O-galactoside; C27 H30 O15; (rt: 40.024)
12
x10 5
117.0339 [C8 H5 O]-
311.0554 [C17 H11 O6]-
431.0973 [C21 H19 O10]-
593.1500 [C27 H30 O15]-
Counts vs. Mass-to-Charge (m/z)

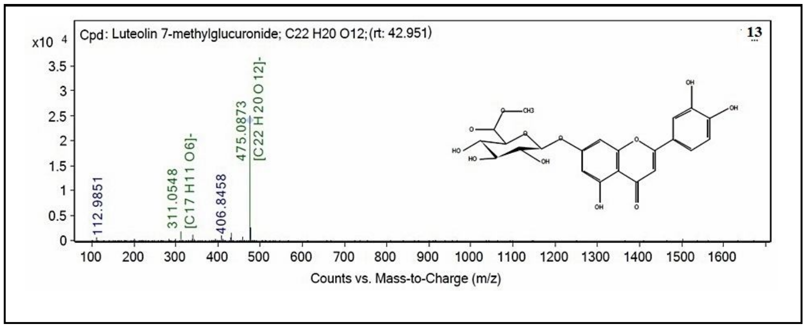

Cpd: Luteolin 7-methylglucuronide; C22 H20 O12; (rt: 42.951)
13
x10 4
112.9851
311.0548 [C17 H11 O6]-
406.8458
475.0873 [C22 H20 O12]-
Counts vs. Mass-to-Charge (m/z)

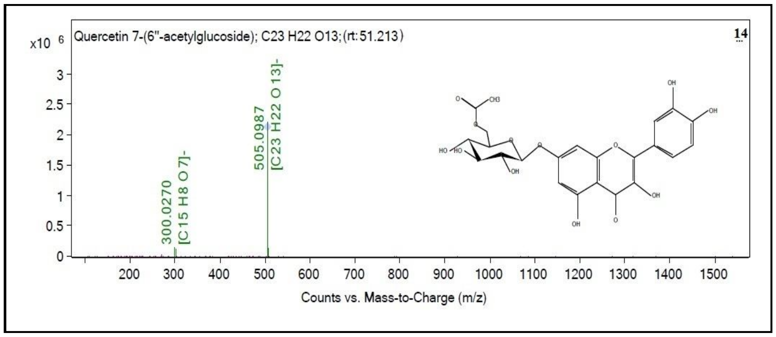

Quercetin 7-(6''-acetylglucoside); C23 H22 O13; (rt:51.213)
14
x10 6
300.0270 [C15 H8 O7]-
505.0987 [C23 H22 O13]-
Counts vs. Mass-to-Charge (m/z)

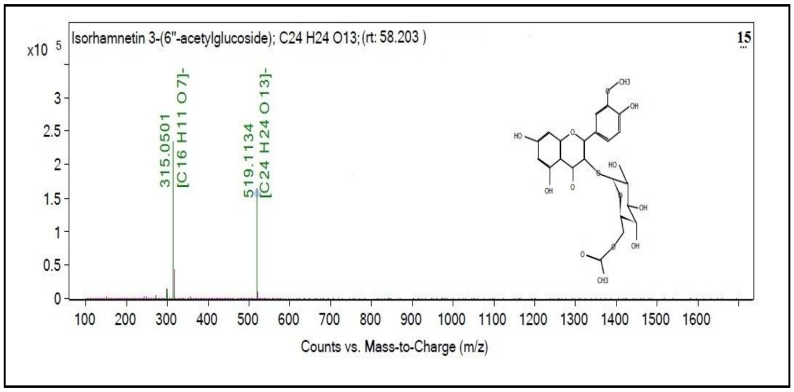

Isorhamnetin 3-(6''-acetylglucoside); C24 H24 O13; (rt: 58.203)
15
x10 5
315.0501 [C16 H11 O7]-
519.1134 [C24 H24 O13]-
Counts vs. Mass-to-Charge (m/z)

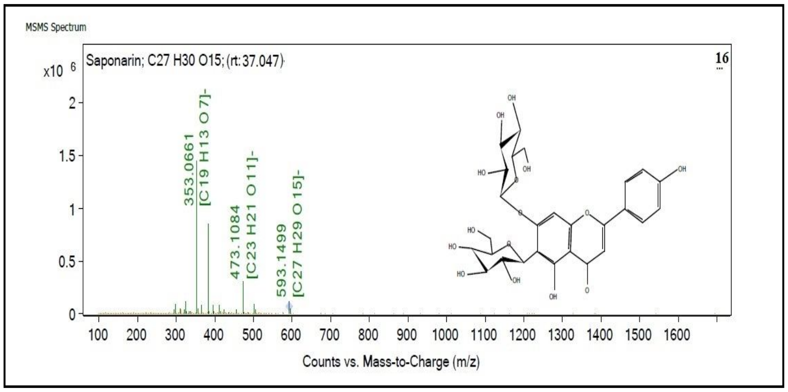

MSMS Spectrum
Saponarin; C27 H30 O15; (rt:37.047)
16
x10 6
353.0661 [C19 H13 O7]-
473.1084 [C23 H21 O11]-
593.1499 [C27 H29 O15]-
Counts vs. Mass-to-Charge (m/z)

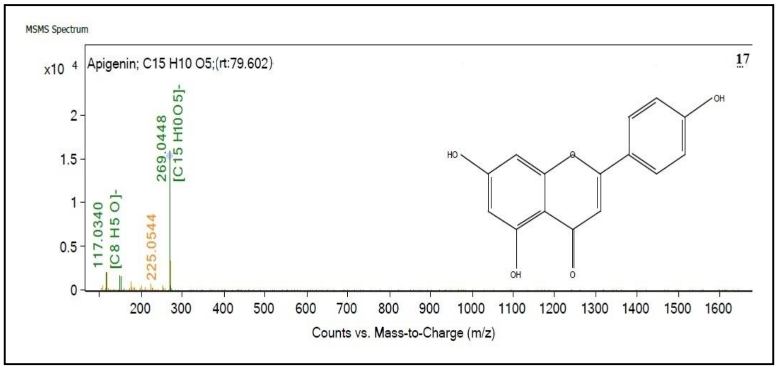
MSMS Spectrum
Apigenin; C15 H10 O5;(rt:79.602)
17
x10 4
117.0340
[C8 H5 O]-
225.0544
269.0448
[C15 H10O5]-
Counts vs. Mass-to-Charge (m/z)

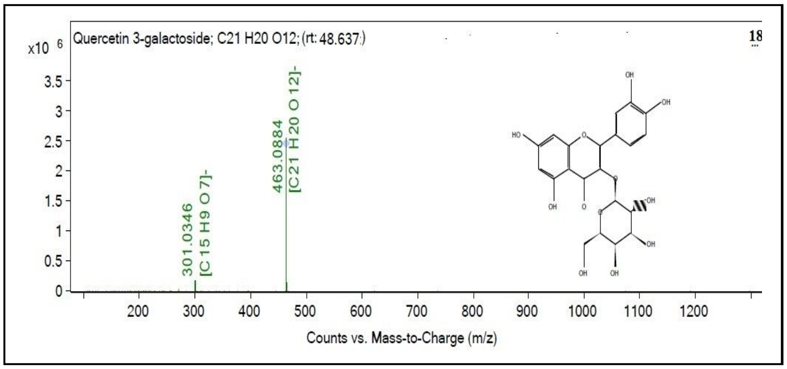
Quercetin 3-galactoside; C21 H20 O12; (rt: 48.637)
18
x10 6
301.0346
[C 15 H 9 O 7]-
463.0884
[C 21 H 20 O 12]-
Counts vs. Mass-to-Charge (m/z)

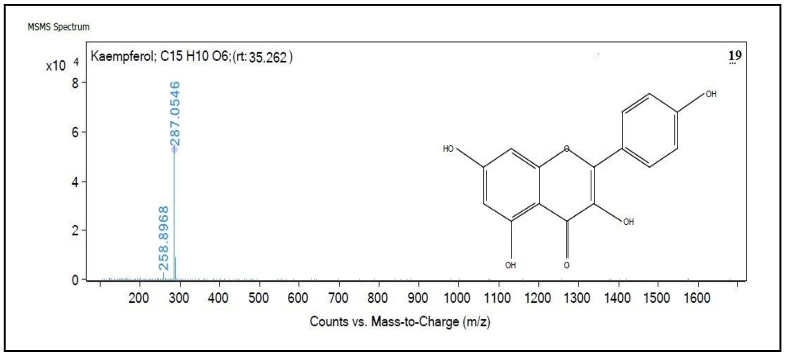
MSMS Spectrum
Kaempferol; C15 H10 O6;(rt: 35.262)
19
x10 4
258.8968
287.0546
Counts vs. Mass-to-Charge (m/z)

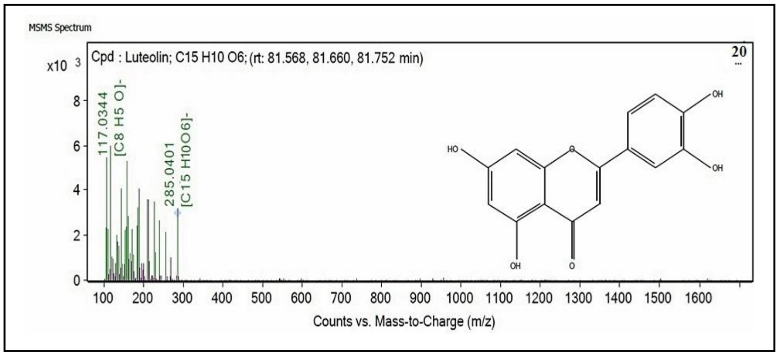
MSMS Spectrum
Cpd : Luteolin; C15 H10 O6; (rt: 81.568, 81.660, 81.752 min)
20
x10 3
117.0344
[C8 H5 O]-
285.0401
[C15 H10O6]-
Counts vs. Mass-to-Charge (m/z)

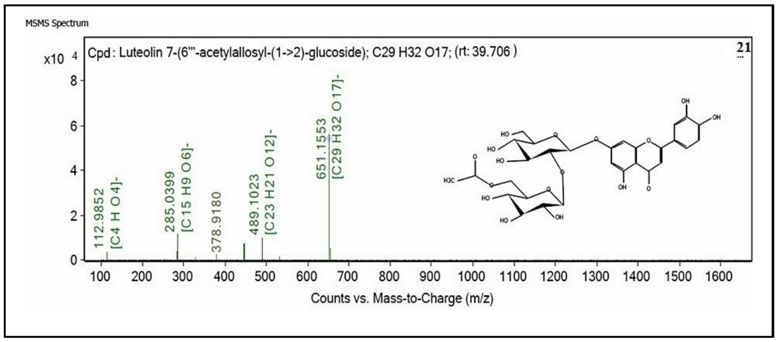
MSMS Spectrum
Cpd : Luteolin 7-(6'''-acetylallosyl-(1->2)-glucoside); C29 H32 O17; (rt: 39.706)
21
x10 4
112.9852
[C4 H O4]-
285.0399
[C15 H9 O6]-
378.9180
489.1023
[C23 H21 O12]-
651.1553
[C29 H32 O17]-
Counts vs. Mass-to-Charge (m/z)

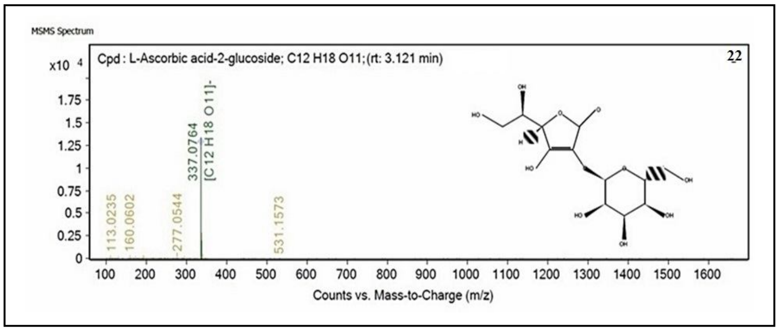
MSMS Spectrum
Cpd : L-Ascorbic acid-2-glucoside; C12 H18 O11; (rt: 3.121 min)
22
x10 4
113.0235
160.0602
277.0544
337.0764
[C12 H18 O 11]-
531.1573
Counts vs. Mass-to-Charge (m/z)

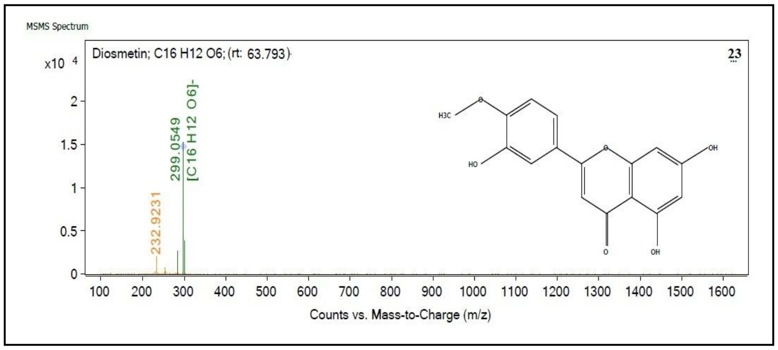
MSMS Spectrum
Diosmetin; C16 H12 O6; (rt: 63.793)
23
x10 4
232.9231
299.0549
[C16 H12 O6]-
Counts vs. Mass-to-Charge (m/z)

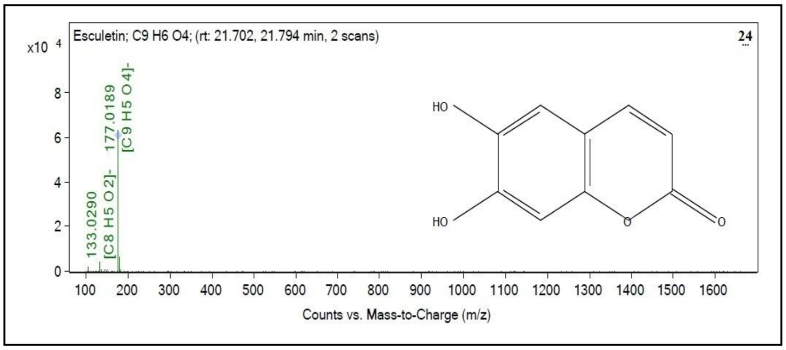
Esculetin; C9 H6 O4; (rt: 21.702, 21.794 min, 2 scans)
24
x10 4
133.0290
[C8 H5 O2]-
177.0189
[C9 H5 O4]-
HO
HO
Counts vs. Mass-to-Charge (m/z)

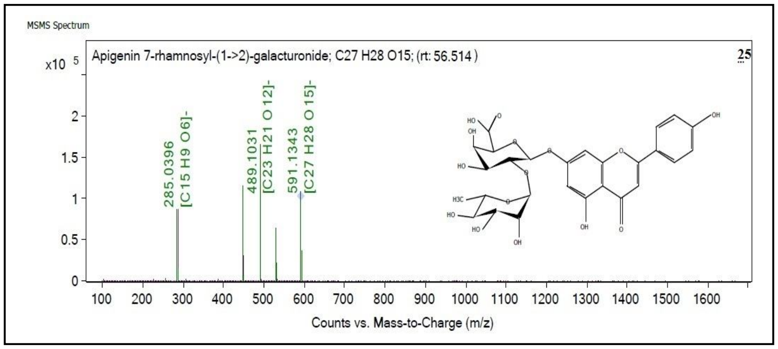
MSMS Spectrum
Apigenin 7-rhamnosyl-(1->2)-galacturonide; C27 H28 O15; (rt: 56.514)
25
x10 5
285.0396
[C15 H9 O6]-
489.1031
[C23 H21 O12]-
591.1343
[C27 H28 O15]-
Counts vs. Mass-to-Charge (m/z)

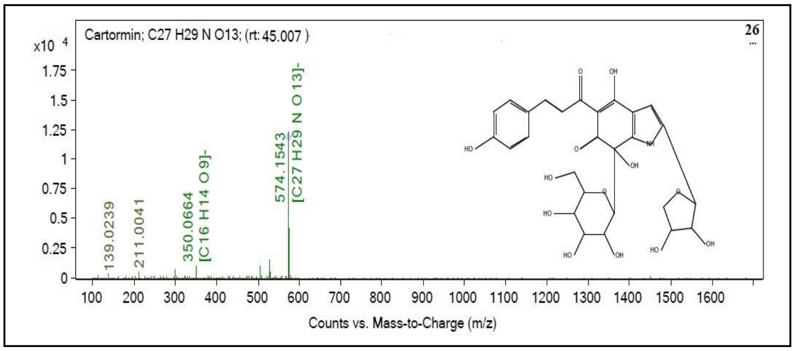
Cartormin; C27 H29 N O13; (rt: 45.007)
26
x10 4
139.0239
211.0041
350.0664
[C16 H14 O9]-
574.1543
[C27 H29 N O13]-
Counts vs. Mass-to-Charge (m/z)

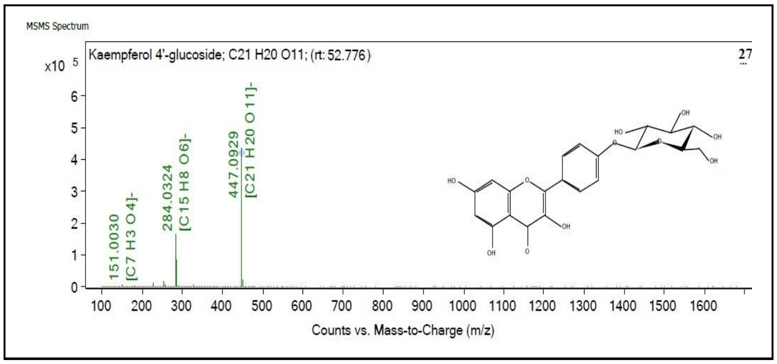
MSMS Spectrum
Kaempferol 4'-glucoside; C21 H20 O11; (rt: 52.776)
27
x10 5
151.0030 [C7 H3 O4]-
284.0324 [C15 H8 O6]-
447.0929 [C21 H20 O11]-
Counts vs. Mass-to-Charge (m/z)

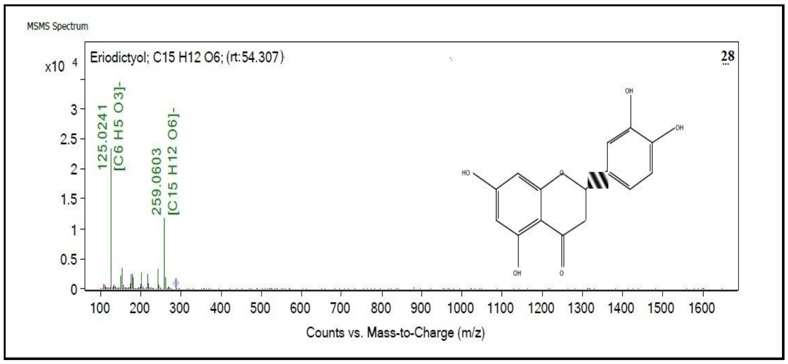
MSMS Spectrum
Eriodictyol; C15 H12 O6; (rt:54.307)
28
x10 4
125.0241 [C6 H5 O3]-
259.0603 [C15 H12 O6]-
Counts vs. Mass-to-Charge (m/z)

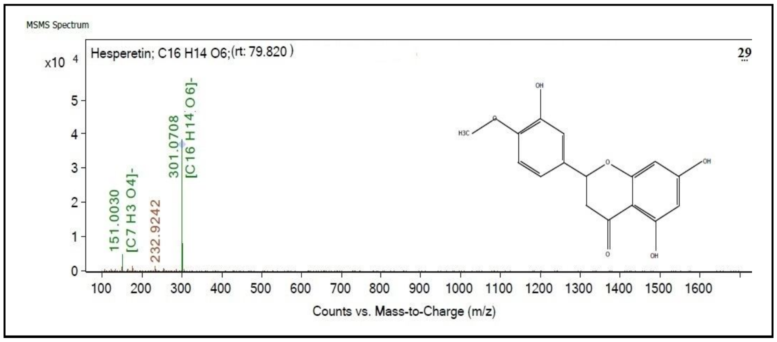
MSMS Spectrum
Hesperetin; C16 H14 O6;(rt: 79.820)
29
x10 4
151.0030 [C7 H3 O4]-
232.9242
301.0708 [C16 H14 O6]-
Counts vs. Mass-to-Charge (m/z)

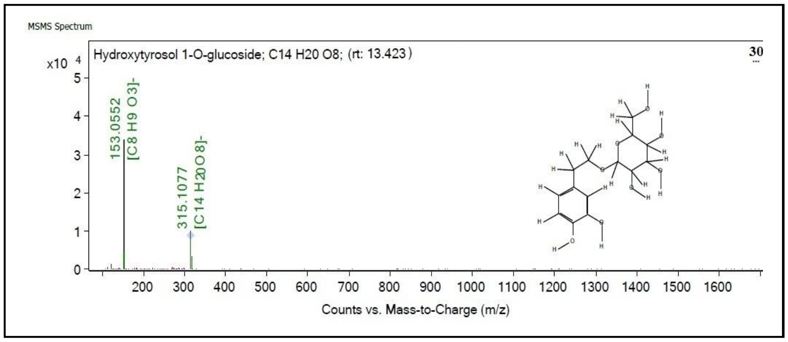
MSMS Spectrum
Hydroxytyrosol 1-O-glucoside; C14 H20 O8; (rt: 13.423)
30
x10 4
153.0552 [C8 H9 O3]-
315.1077 [C14 H20 O8]-
Counts vs. Mass-to-Charge (m/z)

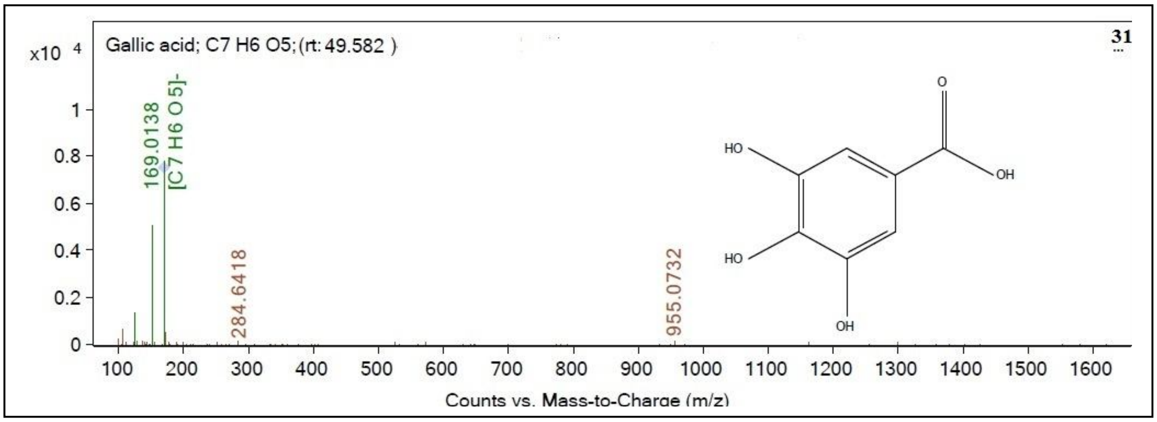
Gallic acid; C7 H6 O5; (rt: 49.582)
31
x10 4
169.0138 [C7 H6 O5]-
284.6418
955.0732
Counts vs. Mass-to-Charge (m/z)
